# Machine learning-based thermoelectric materials screening with physical consistency and uncertainty propagation

**DOI:** 10.1371/journal.pone.0360053

**Published:** 2026-11-02

**Authors:** Abid Ahmad, Mohammad Naved Qureshi, Shabbab Ali Algamdi, Shaha Faisal

**Affiliations:** 1 Research Center for Eco-environmental Engineering, School of Environment and Civil Engineering, Dongguan University of Technology, Dongguan, China; 2 Electrical Engineering Section, University Polytechnic, Aligarh Muslim University, Aligarh, India; 3 Department of Software Engineering, College of Computer Science and Engineering, Prince Sattam bin Abdulaziz University, Al Kharj, Saudi Arabia; 4 College of Business Administration, Prince Sattam bin Abdulaziz University, Al Kharj, Saudi Arabia; Universidad Especializada de las Americas, PANAMA

## Abstract

Machine learning (ML) has emerged as a promising strategy for accelerating thermoelectric materials discovery. However, direct prediction of the thermoelectric figure of merit (ZT) often can introduce target leakage and overestimates predictive performance because ZT is intrinsically derived from multiple transport properties. In this work, a physics-guided and uncertainty-aware ML framework is developed for thermoelectric material screening by independently predicting the Seebeck coefficient, electrical conductivity, and thermal conductivity, followed by analytical evaluation of thermoelectric performance. Experimental data from the ESTM database were evaluated using material-wise validation, with exact chemical formulas kept exclusively in either the training or held-out test subset to reduce material-level information leakage. Predictive uncertainty was quantified using ensemble learning and propagated into thermoelectric performance evaluation and uncertainty-aware LCB screening. The proposed framework demonstrates strong predictive capability for individual transport properties while providing physically consistent thermoelectric performance estimation. Although the framework exhibits lower apparent predictive accuracy than direct ZT regression models, it provides improved interpretability and a more physically structured approach to thermoelectric performance evaluation. The results demonstrate that integrating physical consistency, uncertainty propagation, and uncertainty-aware screening provides a robust strategy for data-driven thermoelectric materials screening.

## Introduction

Thermoelectric materials have attracted considerable attention as sustainable energy-conversion systems because of their ability to directly convert heat into electricity through the Seebeck effect [1,2]. Their solid-state operation, absence of moving components, and scalability make them promising for waste heat recovery, localized cooling, and decentralized energy technologies [3]. Despite these advantages, widespread implementation of thermoelectric devices remains limited by the small number of materials that simultaneously exhibit high conversion efficiency, long-term stability, and practical operating performance across broad temperature ranges [4,5].

The efficiency of thermoelectric materials is commonly evaluated using the dimensionless figure of merit:


$$\begin{array}{c} \text{ZT}=\frac{{\text{S}}^{\text{2}}\sigma \text{T }}{\text{k}}, \end{array}$$ (1)

Where, S, σ, and κ denote the Seebeck coefficient, electrical conductivity, and thermal conductivity respectively, and T is the absolute temperature. Because these transport parameters are strongly interdependent, improving one property often negatively affects another, making rational thermoelectric optimization fundamentally challenging [4,6,7]. Consequently, thermoelectric materials discovery has historically relied on extensive experimental iteration and empirical optimization strategies, resulting in slow and resource-intensive development processes [8–10].

Recent advances in materials informatics and machine learning (ML) have accelerated interest in data-driven approaches for thermoelectric materials discovery [11–13]. Supervised learning models, including random forests, gradient boosting methods, and deep neural networks (DNNs), have demonstrated promising predictive performance for thermoelectric property prediction [14–16]. These approaches offer the potential to rapidly screen large chemical spaces and identify promising candidate materials beyond traditional trial and error experimentation.

Despite these advances, important challenges remain in applying ML to thermoelectric materials discovery. Many previous studies directly predict ZT using compositional descriptors and correlated transport-property information. However, because ZT is mathematically derived from multiple transport quantities, direct regression models can unintentionally exploit hidden statistical correlations within the dataset, leading to target leakage and overly optimistic predictive performance [9,17–20]. In addition, conventional train-test splitting strategies frequently distribute measurements from the same material across both training and testing subsets, which can further inflate predictive accuracy while reducing realistic extrapolative capability for unseen materials [21].

Another limitation of direct ZT-based learning is reduced physical interpretability. Compressing multiple transport phenomena into a single regression target can obscure the competing interactions governing thermoelectric behaviour [20,22]. Recent studies have therefore emphasized the importance of physically consistent ML strategies that independently predict transport properties and subsequently evaluate thermoelectric performance through analytical relationships [22,23]. However, most existing implementations rely primarily on deterministic predictions and do not explicitly propagate uncertainty into thermoelectric performance evaluation.

The importance of uncertainty quantification in scientific machine learning has become increasingly evident, particularly in materials science applications where datasets are sparse, heterogeneous, and experimentally expensive to expand [12,24–26]. Ensemble and Bayesian learning approaches provide practical mechanisms for estimating predictive confidence and distinguishing reliable predictions from uncertain extrapolations. Although uncertainty-aware learning has been explored in thermoelectric materials research, most studies focus on active learning or candidate prioritization without systematically integrating uncertainty propagation into thermoelectric performance assessment [27–30].

To address these challenges, this work presents a physics-guided and uncertainty-aware machine-learning framework for thermoelectric materials discovery. Instead of directly predicting ZT, independent ML models are developed for the Seebeck coefficient, electrical conductivity, and thermal conductivity, followed by analytical thermoelectric performance evaluation. Material-wise validation is employed to ensure strict separation of exact chemical formulas between the training and testing subsets, providing a more realistic assessment of generalization to held-out compositions while reducing material-level information leakage. Predictive uncertainty is quantified using ensemble learning and propagated into thermoelectric performance estimation to obtain uncertainty-aware ZT predictions. Furthermore, lower-confidence-bound (LCB) screening is implemented to prioritize held-out samples by jointly considering predicted performance and associated model uncertainty.

The proposed framework integrates physical consistency, uncertainty propagation, and uncertainty-aware candidate screening within a unified computational materials discovery workflow. By combining independent transport-property prediction with uncertainty-aware thermoelectric evaluation, the framework provides improved interpretability and a more physically structured assessment than conventional direct ZT regression approaches.

## Methodology

### Data source and preprocessing

The thermoelectric dataset used in this study was obtained from the publicly available Experimental Thermoelectric Materials (ESTM) database developed by [31]. The database contains experimentally synthesized thermoelectric materials together with temperature-dependent measurements of key thermoelectric properties, including the Seebeck coefficient, electrical conductivity, thermal conductivity, power factor, and ZT.

Elemental-composition descriptors represented chemical composition and temperature was used as an extra predictor consistent with prior thermoelectric ML studies [15]. Experimentally measured Seebeck coefficient, electrical conductivity, thermal conductivity, power factor, literature reference/DOI, and other source-identifying variables were not included in a predictive feature set used for direct ZT regression, in order to provide a fair and physically transferable comparison between the direct-ZT and the transport-property-based approaches. The direct-ZT baseline was therefore built using the same upstream data that was used for the individual transport-property models: composition-derived descriptors and temperature. We used parameters estimated only from the training data for the continuous predictor variables, which were standardized. Before developing the model, samples with either no target or but nonphysical target values were omitted.

### Learning objective and problem formulation

Unlike conventional approaches that directly regress ZT, the present framework reformulates thermoelectric learning as a transport-property inference problem. Independent supervised learning models were developed for predicting S, σ, and k as functions of composition and temperature. Thermoelectric performance was subsequently evaluated analytically using physically consistent transport relationships rather than direct target regression.

To facilitate the comparison, the predictive inputs were specified uniformly throughout the models. Elemental descriptors derived from the composition and temperatures were used as input features for the direct-ZT regression models. All models were performed with the same composition-derived descriptors in addition to temperature, with the prediction targets being S, σ, and k, respectively. The experimentally measured transport properties, the power factor, the obtained quantities from ZT, literature reference(s) and DOI identifiers were not supplied as predictive inputs and other source-identifying variables.

This strategy reduces the risk of target leakage associated with direct ZT prediction and preserves physically meaningful relationships among transport properties [21]. By independently modeling transport behavior, the framework improves interpretability by retaining the physical relationship between the constituent transport properties and the resulting thermoelectric performance [12,25].

### Data splitting and validation strategy

To ensure a realistic evaluation of predictive performance, data splitting was performed on a material-wise basis rather than by random sampling of individual measurements. The unique characteristics of each material were determined by matching its exact chemical formula as reported in the dataset. All observations with the same precise formula were only assigned to one of the training or test subsets, which includes measurements taken at various temperatures. That is, there was no common formula between the two subsets, limiting the amount of information leakage at material level due to temperature dependent measurements of the same material in both training and testing sets [21]. Different chemical formulas indicating different chemical compositions or concentrations were considered to be different materials. This strategy for validating is therefore a test of generalization to unseen exact compositions, but does not necessarily imply complete extrapolation to entirely new material families.

The unique materials were split into 80% training and 20% held out test. The training subset was used to fit the model, to choose hyper parameters, and to perform cross validation, with the held-out test subset used for final performance evaluation.

### Model architecture and training

Composition-derived elemental descriptors and temperature were chosen as predictive variables for the direct-ZT baseline and evaluated for the Regularized Linear Regression models (Ridge, Lasso, Elastic Net) and the Random Forest regressor. These models were chosen to offer linear, interpretable baselines and nonlinear learning, similar to what has been used in previous studies to predict thermoelectric properties [15,16] using machine learning. The number of trees in the Random Forest model was set to 500 and the random seed was fixed for reproducibility.

The physics-guided framework consisted of training an independent Random Forest regressor for each of the three different properties (Seebeck coefficient, electrical conductivity, and thermal conductivity) on the same composition-derived descriptors and temperature. The electrical conductivity spans a wide dynamic range and was therefore modeled in logarithmic (log10) space and transformed back to the original scale for evaluation and ZT calculation. The Seebeck coefficient and the thermal conductivity were modelled in the original scales.

### Uncertainty estimation via ensemble learning

Five-member Random Forest ensembles have been used to estimate the predictive uncertainty for the transport-property models. The training materials were split into 5 material-wise folds according to the exact chemical formula. Four folds were used for model fitting for each ensemble member and the last “held-out” test set was not used anywhere in the model development. This was done for the Seebeck coefficient, electrical conductivity and thermal conductivity, each by itself. The ensemble mean was adopted as an estimate for the transport-property value for each held-out sample, and the standard deviation of the five ensemble predictions was adopted as an estimate for model-dependent uncertainty of the predictions. This ensemble-based dispersion will give a useful approximation to the uncertainty of the model, but not the total physical or experimental uncertainty of the system [27,28].

### Uncertainty propagation and thermoelectric performance evaluation

Uncertainties associated with predicted transport properties were propagated into thermoelectric performance estimation using first-order error propagation theory. This approach enables probabilistic evaluation of thermoelectric performance by estimating both the expected ZT value and the associated predictive uncertainty interval [32].

Given the predicted mean values$\;\hat{\text{S}}$, $\hat{\sigma }$, $\hat{\text{k}}$ and their associated uncertainties $\sigma_{\text{S}},\sigma_{\sigma },\sigma_{\text{k}}$ the uncertainty in ZT is estimated using first-order error propagation based on a Taylor series expansion. Accordingly, the variance in ZT can be expressed as


$$\begin{array}{c} \sigma_{ZT}^{\text{2}}={\left(\frac{\partial ZT}{\partial S}\right)}^{\text{2}}\sigma_{S}^{\text{2}}+{\left(\frac{\partial ZT}{\partial \sigma }\right)}^{\text{2}}\sigma_{\sigma }^{\text{2}}+{\left(\frac{\partial ZT}{\partial k}\right)}^{\text{2}}\sigma_{k}^{\text{2}} \end{array}$$ (2)

Assuming temperature is treated as an input without an associated predictive uncertainty, the expanded first-order expression is given by:


$$\begin{array}{c} {\;\sigma }_{\text{ZT}}^{\text{2}}={\left(\frac{\text{2}\text{S}\sigma \text{T}}{\text{k}}\right)}^{\text{2}}\sigma_{\text{S}}^{\text{2}}+{\left(\frac{{\text{S}}^{\text{2}}\text{T}}{\text{k}}\right)}^{\text{2}}\sigma_{\sigma }^{\text{2}}+{\left(\frac{{\text{S}}^{\text{2}}\sigma \text{T}}{{\text{k}}^{\text{2}}}\right)}^{\text{2}}\sigma_{\text{k}}^{\text{2}} \end{array}$$ (3)

The first order propagation presented here is valid under the assumption of the statistical independence of the prediction errors of the Seebeck coefficient, the electrical conductivity and thermal conductivity [32]. The approximation does not consider the possible correlations of the predicted transport properties. Thus, the resulting ZT uncertainty is taken to be an approximate model based uncertainty, not a complete representation of experimental, processing, microstructural or other sources of physical variability.

Due to the nonlinear dependency of the transport properties on ZT, the propagation of the uncertainties was also performed by joint ensemble propagation. The ensemble members were used to make predictions of each held out sample’s S, σ, and κ values, which were then combined using the analytical ZT relationship. The average of the obtained ZT ensemble was adopted as the predicted ZT as the model-based predictive uncertainty was adopted as the standard deviation of the resulting ensemble of ZT. This method preserves the nonlinear interaction between the individual transport properties predicted, but does not involve interpretation of the uncertainty obtained as a full physical uncertainty measure. The joint-ensemble standard deviation was used as the primary ZT uncertainty for the subsequent LCB screening, while the first-order propagation formulation was retained as an analytical approximation.

The usefulness of the ensemble uncertainty estimate was checked via the absolute ZT prediction error on the held-out set. There was a positive Spearman correlation between predicted uncertainty and absolute error (ρ = 0.583, p < 0.001). The mean absolute error was monotonically increasing from the lowest uncertainty quartile (0.041) to the highest uncertainty quartile (0.236) and was 0.084 and 0.117 in the latter two quartiles. Thus, the results suggest that a larger ensemble uncertainty leads to a larger prediction error in the held-out data.

### Uncertainty-aware LCB screening

The lower-confidence-bound (LCB) criterion was employed in a screening strategy and the held-out thermoelectric samples ranked. The ensemble models were used to create a measure of predictive uncertainty, which was included in the ranking to give a penalty to samples that predict high thermoelectric performance but have high model uncertainty. One practical approach to scientific machine learning that incorporates model dependent uncertainty is ensemble based predictive uncertainty [27]. The samples were ranked according to the LCB score which was calculated as follows:


$$\begin{array}{c} \text{LCB}=\mu \text{ZT}-\gamma \sigma \text{ZT} \end{array}$$ (4)

The ensemble-averaged predicted ZT is given by μZT, and the propagated predictive uncertainty is σZT while the parameter γ represents risk aversion and is a penalty for uncertain predictions. A value of γ = 1.0 was used for the primary LCB ranking, providing a direct one-standard-deviation penalty for predictive uncertainty. Sensitivity of the ranking was additionally examined for γ = 0, 0.5, 1.0, and 2.0. Thus, samples with high predicted ZT and low predicted uncertainty will be given higher ranks and scores for LCB.

This uncertainty-aware prioritization is now starting to be adopted in materials-informatics and active-learning techniques for minimizing the risk of choosing candidates purely based on uncertain point predictions [29]. The LCB criterion is applied as a retrospective screening/prioritization method on the held-out data set in the present work. Uncertainty that is derived from the ensemble mainly corresponds to model-dependent epistemic uncertainty, and does not fully delineate experimental error, processing conditions, carrier concentrations, microstructural variations or other unobserved physical factors. Thus, the final LCB score is a measure of uncertainty-aware ranking, and not a complete posterior predictive or an absolute measure of total uncertainty.

A sensitivity analysis was performed by varying the risk-aversion parameter over γ = 0, 0.5, 1.0, and 2.0. When no uncertainty penalty was applied γ = 0, Sample 3845 achieved the highest ranking. For γ = 0.5, 1.0, and 2.0, Sample 3559 remained the highest-ranked held-out sample. Increasing γ altered the composition of the remaining top-ranked samples and progressively favored predictions with lower uncertainty. The primary analysis therefore uses γ = 1.0 as an intermediate uncertainty penalty while the sensitivity analysis demonstrates the effect of different risk preferences on the ranking.

## Results

### Dataset characteristics and thermoelectric property distributions

The data used in this study covers a wide temperatures range, from cryogenic temperature to above 1200 K, encompassing both low and high temperature thermoelectric operating regimes. The transport properties show significant variation across different materials. σ spans several orders of magnitude due to differences in carrier concentration and scattering mechanisms. The S includes both positive and negative values, indicating the presence of both n-type and p-type materials. k also varies widely, from ultralow values characteristic of phonon-glass-like systems to higher values associated with crystalline solids. Consequently, ZT ranges from near zero to approximately 2.3, indicating that the dataset includes both low and high-performance thermoelectric materials suitable for machine-learning-based screening. A statistical summary of temperature, transport properties, and thermoelectric performance is provided in Table 1 below.

**Table 1:** Statistical summary of temperature, transport properties, power factor, and ZT in the ESTM dataset.

Variable	Min	Q1	Mean	Q3	Max	Variance
**Temperature (K)**	10	392	539	673	1275	37,026
**Seebeck Coefficient (µV/K)**	−1174	−108	73	202	1052	43,649
**Electrical Conductivity (S/m)**	0.0004	7,059	109,584	91,795	9.46 × 10⁷	2.15 × 1012
**Thermal Conductivity (W/mK)**	0.07	0.73	2.25	2.69	77.16	10.83
**Power Factor (W/mK²)**	0.0000	0.0002	0.0010	0.0013	0.0076	~0.0000
**ZT**	0.00	0.08	0.35	0.53	2.28	0.12

### Machine learning model performance for direct ZT prediction

The predictive performance of the direct-ZT regression models under the material-wise validation strategy is summarized in Table 2. The regularized linear models showed modest predictive performance, with R^2^ values of approximately 0.33. Among the evaluated models, Random Forest achieved the highest performance R^2^ = 0.732, RMSE = 0.163, MAE = 0.104, indicating that nonlinear relationships between composition, temperature, and ZT can be captured more effectively than by the regularized linear baselines. These results provide a leakage-controlled assessment of direct ZT prediction using only composition-derived descriptors and temperature as predictive inputs.

**Table 2:** Performance of direct-ZT regression models using composition-derived elemental descriptors and temperature under material-wise validation.

Model	R²	RMSE	MAE
**Ridge**	0.333	0.257	0.202
**Lasso**	0.339	0.256	0.201
**Elastic Net**	0.338	0.256	0.201
**Random Forest**	**0.732**	**0.163**	**0.104**

### Transport property prediction and statistical relationship with thermoelectric performance

The predictive performance of the independently modeled thermoelectric transport properties is summarized in Table 3. The Seebeck coefficient was predicted with an R^2^ of 0.770, RMSE of 96.181 μV/K, and MAE of 52.384 μV/K. Electrical conductivity showed lower predictive performance (R^2^ = 0.633), RMSE = 57,868.475 S/m, MAE = 29,607.034 S/m), which is consistent with its broad dynamic range and dependence on factors not fully represented by composition and temperature alone. Thermal conductivity achieved an \(R^2\) of 0.841, RMSE of 0.787 W/mK, and MAE of 0.471 W/mK. Because the dataset reports total thermal conductivity without separately resolving lattice and electronic contributions, no phonon-specific interpretation is made. Overall, the results indicate that the composition- and temperature-based models capture a substantial proportion of the variation in the three transport properties, although predictive performance differs across the individual targets.

**Table 3:** Performance of ML models for predicting thermoelectric transport properties using R², RMSE, and MAE.

Property	R²	RMSE	MAE
Seebeck coefficient (μV/K)	0.770	96.181	52.384
Electrical conductivity (S/m)	0.633	57,868.475	29,607.034
Thermal conductivity (W/mK)	0.841	0.787	0.471

### Performance comparison between direct regression and the proposed physics-guided framework

The predictive performance of direct ZT regression and the physics-guided transport-property framework is compared in Table 4. Under the same exact-formula material-wise validation strategy, the direct Random Forest model achieved an R² of 0.732, RMSE of 0.163, and MAE of 0.104. The physics-guided framework, in which ZT was calculated from independently predicted transport properties, achieved an R² of 0.579, RMSE of 0.204, and MAE of 0.119. The direct model therefore provides higher predictive accuracy, whereas the physics-guided framework retains an explicit representation of the underlying transport-property contributions and enables uncertainty propagation through the analytical ZT relationship.

**Table 4:** Performance comparison of direct ZT prediction and the transport-property-based framework using R², RMSE, and MAE.

Approach	Model / Framework	R²	RMSE	MAE
Direct ZT prediction	Random Forest	0.732	0.163	0.104
Physics-guided framework	Transport-property ensemble	0.579	0.204	0.119

### Relationships between transport properties and thermoelectric performance

The relationship between the power factor and the thermoelectric figure of merit shows a clear positive correlation, as presented in Fig 1A. Higher power factor values correspond to higher ZT values, indicating that improving electrical transport properties can enhance thermoelectric performance. However, significant scatter is still observed, particularly at lower power factor values. This dispersion suggests that power factor alone is not sufficient to accurately predict thermoelectric performance. While an increase in power factor often leads to higher ZT, the large spread in the data indicates that other transport parameters, such as k and S, also play important roles in determining overall thermoelectric efficiency.

**Fig 1:**
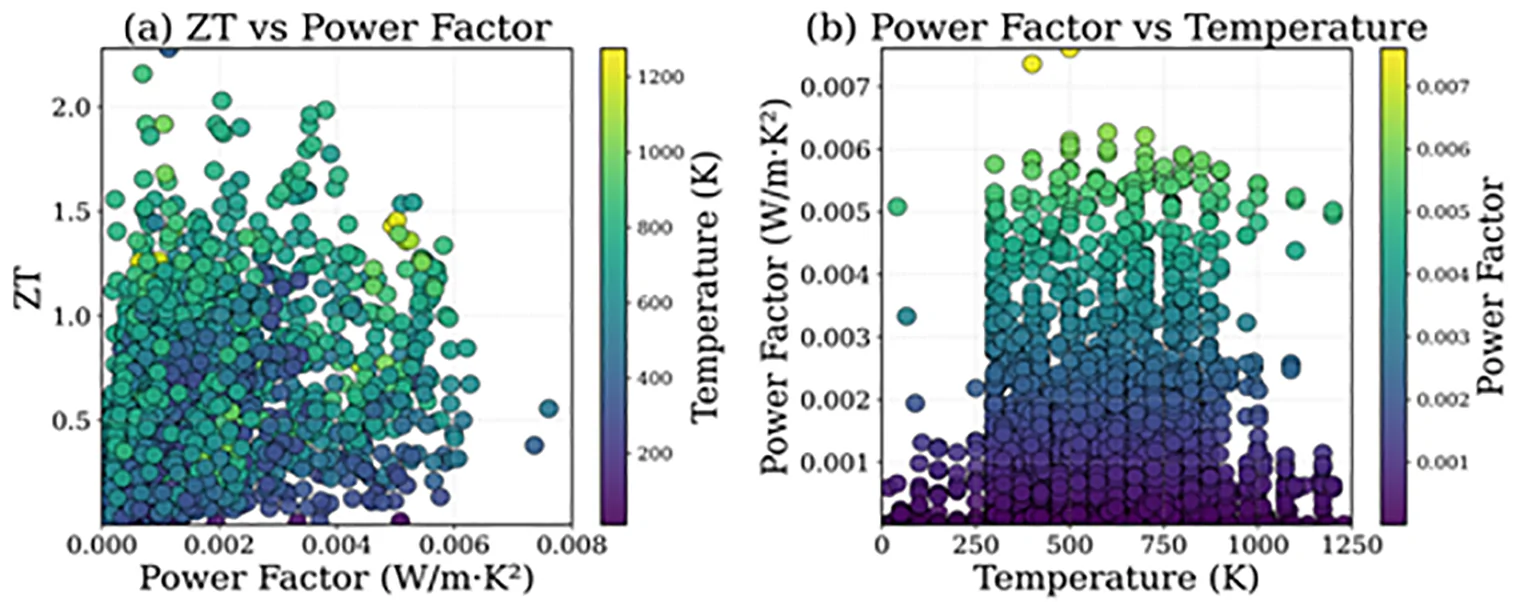
Power factor relationships with ZT and temperature. (A) Relationship between power factor and ZT. (B) Temperature-dependent variation in power factor. The scatter indicates material-dependent variation across the ESTM dataset.

The temperature dependence of the power factor is shown in Fig 1B. The power factor generally increases with temperature, especially in the intermediate to high temperature range. However, a large dispersion is observed at elevated temperatures, indicating strong material dependence and sensitivity to composition and microstructure. This behavior highlights the importance of compositional optimization and materials design for achieving high thermoelectric performance at high temperatures. Overall, although power factor tends to increase with temperature, the performance variation among materials becomes more significant at higher temperatures.

### Model predictions and uncertainty-aware screening

The correlation between predicted and experimental thermoelectric performance is illustrated in Fig 2, where most data points are concentrated along the diagonal reference line, indicating good predictive consistency across a wide range of ZT values. However, increased scatter is observed for higher-ZT samples, indicating greater prediction dispersion in this region of the dataset. The clustering of data points near the diagonal demonstrates agreement between predicted and experimental values, while the increased dispersion at higher ZT values indicates greater prediction-error variability in this region.

**Fig 2:**
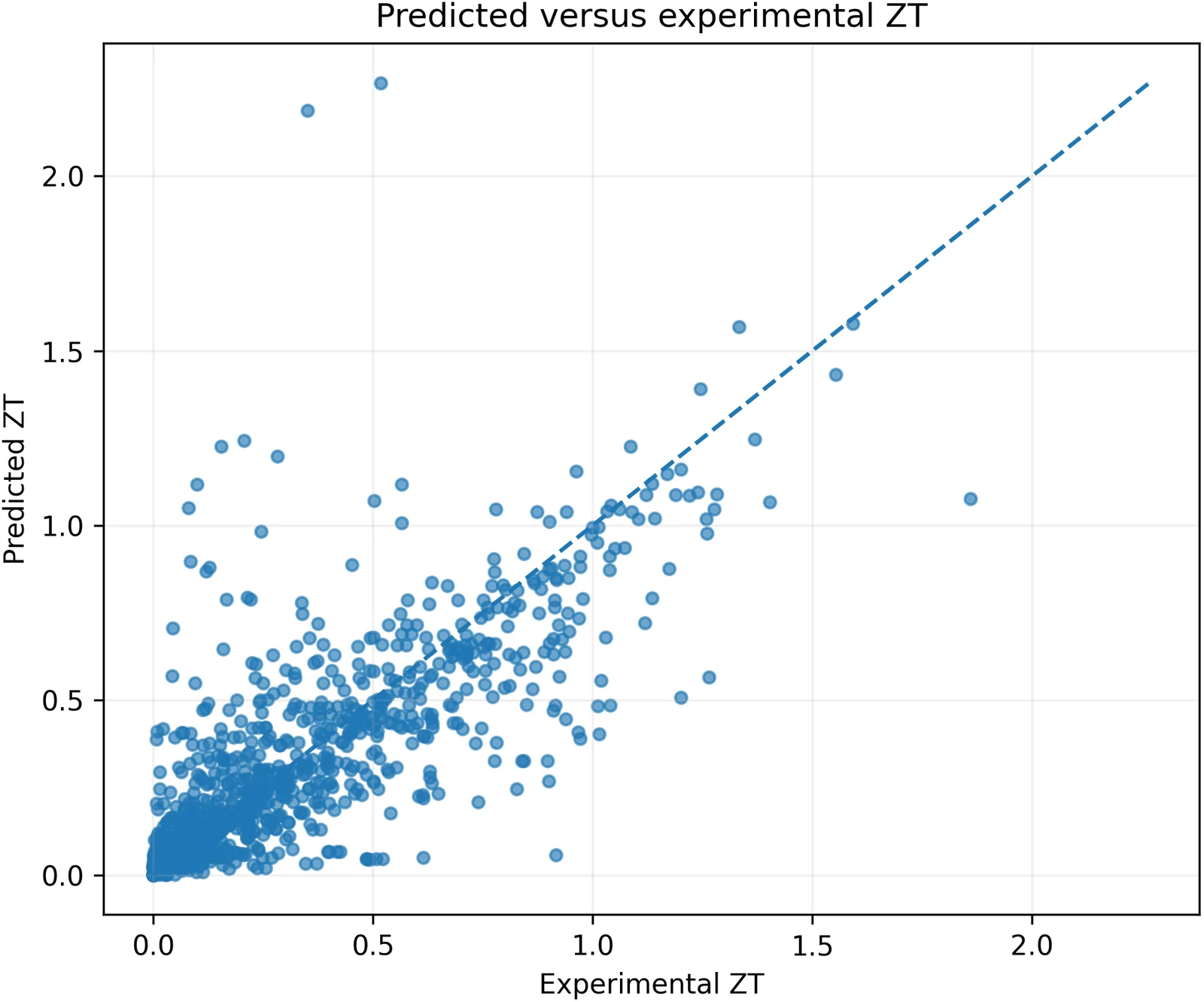
Predicted versus experimental ZT values for the held-out test set. The diagonal reference line indicates ideal agreement between predicted and experimental values.

Table 5 summarizes the highest-ranked held-out thermoelectric samples obtained using the uncertainty-aware LCB criterion. The ranking combines predicted ZT with the corresponding model-based predictive uncertainty, such that samples with high predicted performance but larger uncertainty receive a lower score. Using γ = 1.0, the highest-ranked sample was Sb0.15Ge0.85Te at 823 K (Sample ID 3559), followed by Ge9Sb2Te12 at 827 K (Sample ID 5204). These results represent retrospective screening of held-out samples with experimentally available ZT values and should not be interpreted as the discovery of previously unknown thermoelectric materials.

**Table 5:** Highest-ranked held-out thermoelectric samples obtained using uncertainty-aware LCB screening with γ = 1.0.

Sample ID	Composition	Temperature (K)	Experimental ZT	Predicted ZT	Uncertainty	LCB
3559	Sb0.15Ge0.85Te	823	1.591	1.578	0.200	1.378
5204	Ge9Sb2Te12	827	1.333	1.568	0.200	1.369
3558	Sb0.15Ge0.85Te	773	1.554	1.433	0.269	1.164
1716	Sn0.97Pb0.01 Cd0.025Se	873	1.245	1.390	0.245	1.145
3661	Ge0.6Sb0.2Te0.8(AgSnSe2)0.2	723	1.369	1.247	0.122	1.125

The ranked distribution of held-out thermoelectric samples based on LCB scores is presented in Fig 3, where a small group of materials is clearly separated from the rest, enabling straightforward prioritization for further study. Higher-ranked candidates represent a favorable balance between strong predicted performance and low uncertainty, making them suitable for prioritization for further study.

**Fig 3:**
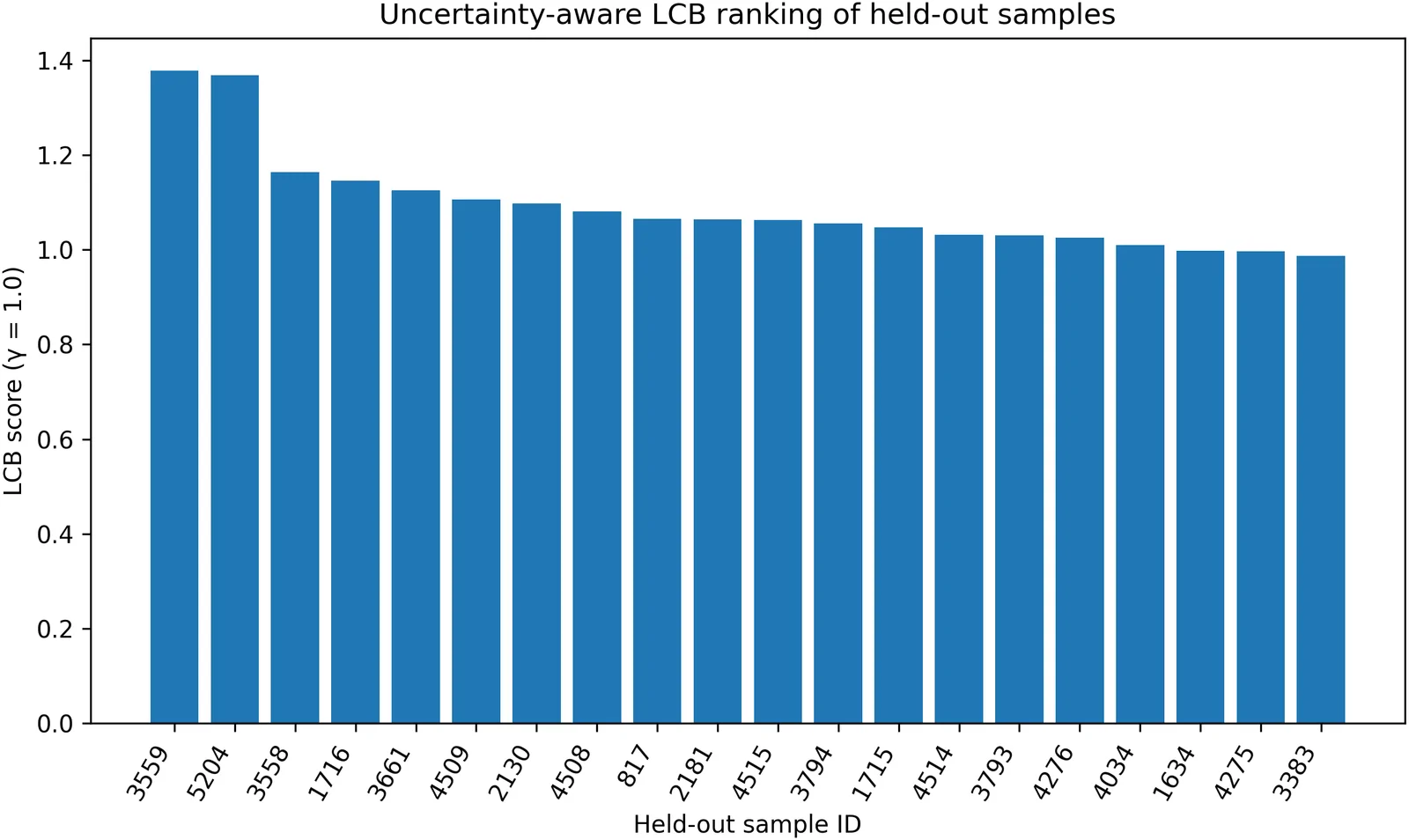
Uncertainty-aware LCB ranking of held-out thermoelectric samples using γ = 1.0. Higher LCB scores indicate a more favorable balance between predicted ZT and model-based predictive uncertainty.

A comparison between predicted and experimental values for key thermoelectric transport properties is presented in Fig 4, including (a) S, (b) σ, and (c) k. In Fig 4A, the predicted S shows strong linear alignment with experimental values, with most data points closely clustered along the diagonal reference line, indicating high predictive accuracy and minimal bias, despite minor deviations at extreme values. Fig 4B illustrates the relationship for σ, where most points also lie near the diagonal, confirming good agreement, although slightly higher dispersion is observed at larger values due to increased variability. Similarly, Fig 4C shows that predicted k values align well with experimental data, with only minor spread at higher ranges reflecting the complexity of thermal transport behavior. Overall, the model demonstrates robust and reliable predictive performance across all three thermoelectric transport properties.

**Fig 4:**
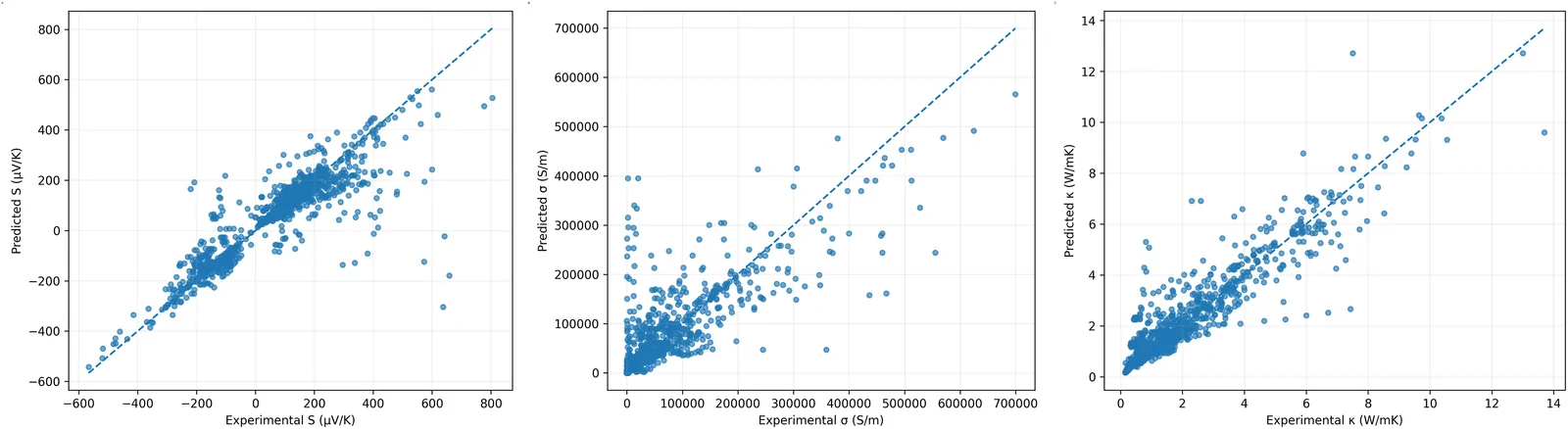
Predicted versus experimental transport properties for the held-out test set. (A) Seebeck coefficient, (B) electrical conductivity, and (C) thermal conductivity. Diagonal reference lines indicate ideal agreement.

## Discussion

### Predictive accuracy and material-wise generalization

The results show the difference between direct ZT regression and estimation of thermoelectric performance using transport properties. As presented in Table 2, the models based on the composition-derived descriptors and temperature are indeed nonlinear models, which perform better for the prediction of direct ZT than the regularized linear models. In these models, Random Forest gives the best prediction accuracy, suggesting that it is possible to effectively capture the nonlinear relationships between composition, temperature and ZT values. Yet, standard ZT regression only considers thermoelectric performance as a single target to be predicted and does not explicitly include the contributions of the transport properties.

The proposed physics-guided approach, on the other hand, characterizes thermoelectric performance based on independently predicted transport properties rather than direct regression of the derived ZT target. The material-wise validation strategy guarantees that each chemical formula is only in one set, either training set or held-out test set, to prevent information leakage from repeated measurement of the same material at different temperature. The resulting evaluation is then an evaluation of generalization to held-out exact compositions. It is not an indication of extrapolation to totally unseen families of material counterparts as there can be chemically similar alloys or doped versions that may be present in both the training and test sets.

### Physical consistency through transport-property inference

The predictive performance of the independently modelled transport properties, reported in Table 3 and validated through Fig 4, demonstrates the effectiveness of the proposed physics-guided framework. By modeling the S, σ, and k separately, thermoelectric performance emerges from realistic trade-offs among transport properties rather than from direct regression of a derived performance metric. This approach preserves physically meaningful relationships among transport parameters and is consistent with thermoelectric transport theory and previous studies (12, 25).

Electrical and thermal transport behavior combine to control the thermoelectric performance, which cannot be described by a single parameter. Power factor, temperature and thermal conductivity are directly related to ZT, and the influence of Seebeck coefficient and electrical conductivity are indirectly related to other transport quantities. The proposed framework maintains this physical structure, while also independently predicting the constituent transport properties and estimating the thermoelectric performance using an analytical relationship.

### Role of uncertainty quantification in thermoelectric materials screening

Uncertainty estimates for predictions of transport properties are more than deterministic point estimates. An ensemble-based predictive uncertainty measure had a positive correlation with the observed absolute ZT prediction error (Spearman’s ρ = 0.583), (p < 0.001) in the held-out test set. The mean absolute error worsened with uncertainty quartile, from 0.041 in the lowest uncertainty quartile to 0.084, 0.117 and 0.236 in the highest uncertainty quartile. This correlation suggests that the ensemble uncertainty estimate is useful to pinpoint predictions with a higher probability of being more uncertain.

The uncertainty reported, however, is largely model-dependent. It cannot completely explain the experimental measurement error, the processing history, the carrier concentration, microstructural variation, and other unobserved physical factors. Therefore, the propagated ZT uncertainty should be viewed as an approximation of model-based uncertainty (and not as a physical uncertainty). In this context, the uncertainty estimate is used to prioritize held out samples using the LCB criterion, instead of just using the predicted ZT.

### Uncertainty-aware LCB screening for risk-aware prioritization

To demonstrate the effectiveness of uncertainty-aware LCB screening, we show the ranked held-out samples in Table 5 and the ranked distribution in Fig 3. The LCB approach is a compromise between the expected performance and predictive uncertainty, favoring samples with high expected performance and relatively high prediction confidence. This helps to minimize the chance of selecting samples with relatively high predictive uncertainty.

Uncertainty-aware screening strategies have become more and more important in materials informatics applications [29,30] and can be incorporated into thermoelectric materials screening to effectively prioritize held-out samples. The results illustrate that uncertainty-sensitive ranking is more informative for sample prioritization than simply ranking based on predicted performance.

### Methodological contributions and broader impact

The proposed framework is tested in three ways: first, by evaluating the framework for transport-property prediction (Table 3); second, by comparing the framework to direct ZT regression (Table 4); and third, by screening held out samples using the proposed framework with uncertainty (Table 5). The framework does not depend solely on the direct regression of ZT, but also relies on the prediction of the component transport properties by itself, and then the assessment of the thermoelectric performance based on the analytical relationship. This formulation allows for the propagation of model based predictive uncertainty to ZT and the incorporation of the same in sample prioritization based on LCB.

A key methodological advance is thus the seamless coupling of transport-property inference, physically structured ZT evaluation, uncertainty propagation, and uncertainty-aware screening in a single machine-learning pipeline. The method provides an interpretable framework for thermoelectric performance estimation and retrospective screening under material-wise validation, subject to the descriptors and experimental information available in the dataset.

### Limitations and future directions

The proposed framework has some drawbacks given the size and diversity of the available experimental thermoelectric dataset. Furthermore, the thermoelectric transport behavior is not just determined by composition and temperature. The Seebeck coefficient, as well as electrical and thermal conductivity and ZT, are physical properties which may significantly depend on the carrier concentration, doping level, defect structure, phase constitution, grain structure, microstructure, synthesis route and processing conditions. These variables are not always available in the current dataset, so they should not be considered as simply noise, but instead as the loss of physical information. The absence of these variables may increase prediction error and limits the extent to which the reported model uncertainty can represent the total experimental and physical variability of thermoelectric systems. In future, these materials descriptors, processing information, physics-informed constraints, and family-level validation (where sufficient data exists) should be included.

Nevertheless, the results demonstrate that incorporating physical consistency and uncertainty quantification leads to more reliable and scientifically meaningful predictions compared to purely accuracy-driven machine learning models. The framework therefore provides a practical pathway toward more reliable machine-learning-guided thermoelectric materials discovery.

## Conclusion

This paper describes a physics-guided and uncertainty-aware machine-learning-based thermoelectric performance estimation approach. The framework does not directly predict ZT from the experimentally measured transport quantities, but instead uses temperature and composition-derived descriptors to independently model the Seebeck coefficient, electrical conductivity, and thermal conductivity, from which ZT is calculated from its analytical relationship. To minimize repeated measurement of the exact composition, the information leakage associated with repeated measurements is reduced by material-wise validation.

Ensemble-based uncertainty estimation and first-order uncertainty propagation allows the uncertainties of a model to be fed-back into thermoelectric performance evaluation. Uncertainty-aware LCB screening also offers an intuitive way of prioritizing held-out samples, simultaneously accounting for expected ZT and predictive uncertainty. The direct-ZT Random Forest model achieved higher predictive accuracy than the physics-guided framework, whereas the physics-guided approach provides a more interpretable and physically structured pathway by explicitly modeling the constituent transport properties and enabling uncertainty propagation.

The framework is not comprehensive because of limited descriptors of composition and temperature and is limited in its ability to represent processing history, carrier concentration, microstructure, and other material-specific factors that affect thermoelectric performance. However, the study illustrates the viability of the integrated approach of transport property inference, physical relationships, and uncertainty modeling to enable more transparent thermoelectric materials screening.
